# Lifestyle, Inflammation, and Periodontitis: A National Study Based on the Life’s Crucial 9 Framework

**DOI:** 10.3290/j.ohpd.c_2312

**Published:** 2025-10-20

**Authors:** Baolin Jia, Qiang Wang, Jun Ren, Guixin Li, Xianjie Zheng, Sen Yang

**Affiliations:** a Baolin Jia Attending Physician, Department of Oral and Maxillofacial Surgery, Suining Central Hospital, Suining, Sichuan, China. Conceptualization, methodology, formal analysis, data curation, wrote the original draft.; b Qiang Wang Associate Chief Physician, Department of Oral and Maxillofacial Surgery, Suining Central Hospital, Suining, Sichuan, China. Investigation, software, resources, wrote the original draft.; c Jun Ren Attending Physician, Department of Oral and Maxillofacial Surgery, Suining Central Hospital, Suining, Sichuan, China. Data curation, validation, visualisation, reviewed and edited the manuscript.; d Guixin Li Resident Physician, Department of Oral and Maxillofacial Surgery, Suining Central Hospital, Suining, Sichuan, China. Methodology, supervision, project administration, reviewed and edited the manuscript.; e Xianjie Zheng Resident Physician, Department of Oral and Maxillofacial Surgery, Suining Central Hospital, Suining, Sichuan, China. Formal analysis, funding acquisition, investigation, reviewed and edited the manuscript.; f Sen Yang Chief Physician, Department of Oral and Maxillofacial Surgery, Suining Central Hospital, Suining, Sichuan, China. Conceptualization, supervision, project administration, reviewed and edited the manuscript.

**Keywords:** LC9, mediation analysis, NHANES, oral health, periodontitis.

## Abstract

**Purpose:**

To examine the association between the Life’s Crucial 9 (LC9) lifestyle score and periodontitis severity among U.S. adults, and to explore the mediating roles of systemic inflammation and oxidative stress. The study hypothesis is that higher LC9 scores are associated with lower periodontitis severity.

**Materials and Methods:**

This cross-sectional study used data from 7124 adults aged ≥30 years from the 2009–2014 National Health and Nutrition Examination Survey (NHANES). Weighted logistic, ordinal logistic, and linear regression models assessed the relationships between LC9 and periodontitis risk, severity, clinical attachment loss (CAL), and probing depth (PD). Restricted cubic spline (RCS) regression examined dose–response trends. Weighted quantile sum (WQS) regression evaluated the relative contribution of LC9 components. Mediation analysis was performed to assess inflammatory and oxidative pathways. Receiver operating characteristic (ROC) analysis compared the predictive performance of LC9 and Life’s Essential 8 (LE8).

**Results:**

Each 10-point increase in LC9 was associated with a reduced risk of periodontitis (OR = 0.858, 95% CI: 0.808–0.911), milder severity, and lower CAL and PD. Tobacco exposure and glycemic control were the strongest contributors. White blood cell count, systemic inflammation index (SIRI), and albumin mediated 25.1%, 7.3%, and 5.3% of the LC9–periodontitis relationship, respectively. No statistically significant mediation was observed for oxidative stress. LC9 and LE8 demonstrated comparable predictive performance (AUC: 0.758 vs 0.759).

**Conclusion:**

Higher LC9 scores were statistically significantly associated with better periodontal outcomes, primarily through inflammatory pathways. Clinically, modifying key lifestyle factors — especially tobacco avoidance and glycemic control — may effectively reduce periodontitis risk.

In the United States, periodontitis is a widespread chronic inflammatory disease. It is is not contagious, and both environmental and genetic factors contribute to its development.^28^ It presents a major challenge to public health, not only due to its high prevalence but also because of its substantial economic and societal burden.^27^ It affects approximately 42.2% of individuals.^4^ In addition to inducing tooth mobility and compromised masticatory function, periodontitis is statistically significantly associated with numerous systemic illnesses, including cardiovascular disease (CVD).^19,23^ The pathogenic pathways are thought to facilitate the initiation and advancement of systemic diseases by fostering low-grade systemic inflammation and atherosclerosis.^2,24^ Therefore, identifying modified lifestyle factors is crucial for the prevention and management of periodontitis and for alleviating its systemic effects, particularly those linked to cardiovascular disease, which present substantial public health challenges.^6^

Growing evidence points to a two-way link between cardiovascular health and periodontal disease.^21,30,31^ The American Heart Association recently unveiled the LC9, an enhancement of the LE8, by integrating a mental health element — specifically depression — into the lifestyle evaluation framework.^8,32^ This revised model seeks to deliver a more thorough assessment of overall health by incorporating both behavioral and psychological factors pertinent to chronic disease risk.^33^

Prior research has demonstrated an association between depression and periodontitis, probably due to the combined effects of inflammation and poor dental care. Moreover, elevated LE8 scores are associated with a lower likelihood of periodontitis. This relationship has been confirmed by multiple studies using NHANES data.^18^ To the best of the authors’ knowledge, no study has comprehensively explored the link between LC9, particularly its mental health component, and the severity of periodontitis. Furthermore, the specific contributions of each LC9 component to this association remain unquantified.

Periodontitis follows a well-documented natural history, as demonstrated by the landmark Sri Lanka and Oslo studies, which highlighted its progressive nature in the absence of preventive or therapeutic interventions.^17^ Moreover, inflammation and oxidative stress are acknowledged as fundamental pathological mechanisms common to both periodontitis and cardiovascular disease. Elevated systemic inflammatory markers are commonly found in patients with periodontitis. These markers are closely associated with an increased risk of cardiovascular disease.^26^ However, few empirical studies have examined how much these biological mechanisms mediate the link between LC9 and periodontitis.

This study sought to investigate the association between LC9 and periodontitis severity using nationally representative data from NHANES 2009–2014. We hypothesized that elevated LC9 scores would be statistically significantly correlated with reduced severity of periodontitis. Additionally, we proposed that pathways involving systemic inflammation and oxidative stress could partially mediate this relationship.

## MATERIALS AND METHODS

### Ethical Approval and Consent to Participate

This study used publicly available and de-identified data from the National Health and Nutrition Examination Survey (NHANES), which is conducted by the National Center for Health Statistics (NCHS). All NHANES protocols were approved by the NCHS Research Ethics Review Board, and all participants provided informed consent. Therefore, additional ethical approval was not required for this secondary data analysis.

### Study Population

This study analyzed data from the National Health and Nutrition Examination Survey (NHANES) collected between 2009 and 2014. NHANES is an ongoing cross-sectional survey designed to represent the health and nutritional status of the non-institutionalized U.S. population. The survey uses a stratified, multistage probability sampling approach to achieve national representativeness.^1^ Approval for the study was granted by the Ethics Review Board of the National Center for Health Statistics. Written informed consent was obtained from all participants. The study followed the STROBE reporting standards.^41^

Out of the initial 30,468 participants, 19,785 were excluded because of missing or inadequate data regarding periodontitis grading. 2097 participants who lacked LC9-related data were excluded. Furthermore, 1462 individuals were excluded due to missing covariate information. Ultimately, 7124 adults aged 30 years or older were included in the analysis, with 3555 males and 3569 females. Figure 1 illustrates the participant selection process.

### Periodontitis Definition and Classification

Individuals aged 30 years or older possessing at least one permanent tooth were qualified for a periodontal assessment at a mobile examination center (MEC). Periodontal evaluations were performed utilizing a standardized probe to determine gingival recession and probing depth (PD) at six locations per tooth, which were subsequently employed to compute clinical attachment loss (CAL).

Periodontitis was categorized based on the 2012 case definition established by the Centers for Disease Control and Prevention (CDC) and the American Academy of Periodontology (AAP).^5,22^ Periodontitis was categorized as “mild,” “moderate,” or “severe,” and participants who did not meet any of the criteria were classified as having “no periodontitis”.

### Measurement of LC9

The LC9 was calculated by averaging the eight components of LE8 with the depression score.^40^ Depression scores were obtained from the Patient Health Questionnaire-9 (PHQ-9) and categorized into scores of 100, 75, 50, 25, and 0, corresponding to PHQ-9 total score ranges of 0–4, 5–9, 10–14, 15–19, and 20–27, respectively.^16^ The LE8 score encompasses diet, physical activity, nicotine exposure, sleep, as well as body mass index (BMI), blood lipids, blood glucose, and blood pressure. Dietary intake was evaluated through a 24-hour dietary recall and scored based on the Healthy Eating Index 2015 (HEI-2015).^15^ Physical activity, smoking behavior, sleep duration, and history of diabetes were collected via standardized questionnaires, while blood lipids, glucose levels, BMI, and blood pressure were measured through laboratory tests and physical examinations.

### Covariates and Mediating Variables

Demographic covariates were acquired using household questionnaires. Race/ethnicity was categorized as Mexican American, non-Hispanic Black, non-Hispanic White, other Hispanic, and other races.

Educational attainment was categorized as less than high school, high school, and above high school. The drinking status was categorized as never, light, moderate, heavy, and former drinkers. Oral hygiene practices were evaluated as follows: Flossing behavior was quantified by the frequency of floss or flossing equipment usage during the preceding 7 days, with 1–7 days categorised as “yes” and 0 days as “no”. Mouthwash use was determined based on self-reported activity during the past seven days (yes/no). The chosen covariates aimed to augment the LC9 score, which already encompasses data on dietary quality, duration of physical activity, smoking habits, sleep duration, body mass index, blood lipids, blood glucose, and blood pressure.

In the mediation analysis, white blood cell count (WBC), albumin (g/dL) and systemic inflammatory response index (SIRI) served as indices of systemic inflammation. Gamma-glutamyl transpeptidase (GGT, U/L), bilirubin (mg/dL), and uric acid (mg/dL) were incorporated as biomarkers of oxidative stress. The calculation of SIRI was performed using the formula: SIRI = (platelet count × neutrophil count) / lymphocyte count.^34,36^

### Statistical Analysis 

All analyses were conducted using R software version 4.4.3 (Vienna, Austria), applying complex survey design and weights (SDMVSTRA, SDMVPSU, WTMEC2YR) according to NHANES guidelines.

Continuous variables were expressed as weighted means ± standard deviations (SD) and compared using weighted t-tests. Categorical variables were expressed as weighted percentages and analyzed with weighted chi-squared tests.

Weighted multivariable logistic regression was utilized to examine the association between LC9 and periodontitis prevalence, while ordered logistic regression was employed to assess disease severity. Additionally, weighted linear regression analyzed CAL and PD.Three models were constructed: Model 1: unadjusted; Model 2: adjusted for age, sex, race, education, marital status, and PIR; Model 3: further adjusted for drinking status, flossing, and mouthwash use.

Dose-response relationships were assessed using trend tests and restricted cubic splines (RCS). The predictive performance of LC9 compared to LE8 was evaluated by calculating receiver operating characteristic (ROC) curves and area under the curve (AUC).

Subgroup and interaction analyses were stratified by sex, age, race, education, PIR, marital status, drinking status, flossing, and mouthwash use.

All nine LC9 components were constructed as ordinal variables and entered into weighted quantile sum (WQS) regression to assess their relative importance, preserving their clinical gradient structure.

Mediation analysis was conducted utilizing the “mediation” package in R. The bootstrap technique (1000 iterations) was employed to assess the indirect effects of systemic inflammation and oxidative stress within the LC9–periodontitis pathway.

## RESULTS

The data collected here can be found in the National Health and Nutrition Examination Survey.^5^

### Participant Demographics 

Among 7124 participants, 3504 had periodontitis and 3620 did not. Those with periodontitis were older (55.01 vs 48.79 years), more often male (58.8% vs 43.8%), and had lower socioeconomic status (PIR: 2.78 vs 3.59) (all p < 0.001). They also showed lower prevalence of flossing (67.7% vs 78.3%) and lower scores for glycemic control (79.74 vs 88.68), lipid profile (58.98 vs 62.12), and blood pressure (63.00 vs 72.61) (all p < 0.001). Systemic inflammation and oxidative stress markers were higher in the periodontitis group, including SIRI (1.33 vs 1.17), WBC (7.33 vs 6.85 ×10^3^/µl), GGT (30.64 vs 26.10 U/l), and uric acid (5.59 vs 5.34 mg/dl) (all p < 0.01). Bilirubin and PHQ-9 scores did not differ significantly (p = 0.169 and 0.101, respectively) (Table 1).

**Table 1:** Baseline characteristics of participants by periodontitis status

Characteristic	Total n = 7174	No periodontitis n = 3620	With periodontitis n = 3504	p-value
Age, mean (SD)	51.31 ± 13.34	48.79 ± 12.70	55.01 ± 13.39	<0.001
**Sex, n (%)**				<0.001
Female	3,569 (50.09%)	2,124 (56.18%)	1,445 (41.18%)	
Male	3,555 (49.91%)	1,496 (43.82%)	2,059 (58.82%)	
**Race, n (%)**				<0.001
Mexican American	951 (7.11%)	356 (4.97%)	595 (10.23%)	
Non-Hispanic Black	1,373 (9.31%)	550 (6.95%)	823 (12.76%)	
Non-Hispanic White	3,438 (73.17%)	1,976 (78.23%)	1,462 (65.77%)	
Other Hispanic	645 (4.56%)	324 (4.11%)	321 (5.21%)	
Other Race	717 (5.85%)	414 (5.73%)	303 (6.04%)	
**Marital status, n (%)**				<0.001
Married/living with partner	4,684 (70.53%)	2,498 (74.54%)	2,186 (64.67%)	
Never married	777 (9.49%)	413 (9.07%)	364 (10.11%)	
Widowed/divorced/separated	1,663 (19.97%)	709 (16.39%)	954 (25.22%)	
PIR, mean (SD)	3.26 ± 1.60	3.59 ± 1.52	2.78 ± 1.60	<0.001
Flossing behavior, n (%)	4,983 (73.99%)	2,754 (78.28%)	2,229 (67.70%)	<0.001
Mouthwash behavior, n (%)	4,007 (52.81%)	1,953 (50.70%)	2,054 (55.91%)	0.002
**Education, n (%)**				<0.001
Above high school	4,162 (66.37%)	2,527 (75.40%)	1,635 (53.15%)	
Below high school	528 (3.76%)	155 (2.00%)	373 (6.34%)	
High school	2,434 (29.87%)	938 (22.60%)	1,496 (40.51%)	
**Drinking status, n (%)**				<0.001
Former	1,243 (14.60%)	506 (11.82%)	737 (18.67%)	
Heavy	1,256 (17.54%)	567 (15.57%)	689 (20.43%)	
Mild	2,640 (40.26%)	1,454 (43.22%)	1,186 (35.92%)	
Moderate	1,115 (18.18%)	670 (20.20%)	445 (15.22%)	
Never	870 (9.42%)	423 (9.19%)	447 (9.76%)	
LC9, mean (SD)	71.34 ± 13.23	73.80 ± 12.78	67.74 ± 13.07	<0.001
HEI-2015 diet score, mean (SD)	43.89 ± 31.67	45.43 ± 31.98	41.63 ± 31.08	0.001
Physical activity score, mean (SD)	73.93 ± 40.54	75.60 ± 39.31	71.49 ± 42.16	0.001
Tobacco/nicotine exposure score, mean (SD)	74.13 ± 36.62	80.97 ± 31.87	64.13 ± 40.62	<0.001
Sleep health score, mean (SD)	83.73 ± 23.82	85.31 ± 22.62	81.42 ± 25.31	<0.001
BMI score, mean (SD)	59.64 ± 32.82	60.88 ± 32.53	57.82 ± 33.16	0.002
Blood lipids score, mean (SD)	60.85 ± 30.20	62.12 ± 29.96	58.98 ± 30.45	0.001
Blood glucose score, mean (SD)	85.05 ± 24.32	88.68 ± 21.49	79.74 ± 27.09	<0.001
Blood pressure score, mean (SD)	68.71 ± 30.38	72.61 ± 29.46	63.00 ± 30.81	<0.001
PHQ-9 score, mean (SD)	92.13 ± 18.10	92.58 ± 17.76	91.47 ± 18.58	0.101
Clinical attachment loss (mm), mean±SD	1.61 ± 0.99	1.13 ± 0.36	2.32 ± 1.19	<0.001
Probing pocket depth (mm), mean±SD	1.41 ± 0.56	1.14 ± 0.31	1.80 ± 0.61	<0.001
GGT(U/L), mean±SD	27.94 ± 41.11	26.10 ± 42.78	30.64 ± 38.40	0.002
Bilirubin(mg/dL), mean±SD	0.71 ± 0.29	0.72 ± 0.29	0.71 ± 0.30	0.169
Uric acid(mg/dL), mean±SD	5.44 ± 1.38	5.34 ± 1.36	5.59 ± 1.40	<0.001
Albumin(g/dL), mean±SD	4.28 ± 0.31	4.31 ± 0.31	4.25 ± 0.31	<0.001
White blood cell (1000 cell/uL), mean±SD	7.04 ± 2.18	6.85 ± 1.96	7.33 ± 2.45	<0.001
SIRI, mean±SD	1.23 ± 0.85	1.17 ± 0.80	1.33 ± 0.92	<0.001
Note: Continuous variables are presented as mean ± standard deviation (SD); categorical variables as number (weighted %). Abbreviations: BMI, body mass index; PIR, poverty income ratio; HEI, Healthy Eating Index; PHQ-9, Patient Health Questionnaire-9; SIRI, systemic inflammation response index.

### Weighted Logistic Regression Analysis of LC9 and Periodontitis Prevalence

After full adjustment (Model 3), each 10-point increase in LC9 was associated with lower odds of periodontitis (OR = 0.858, 95% CI: 0.808–0.911, p < 0.001). Compared with the lowest quartile (Q1), participants in the highest quartile (Q4) had an approximately 35% lower risk (p for trend < 0.001), indicating a clear dose-response relationship (Table 2). Further analyses of periodontitis severity and continuous periodontal measures (CAL and PD) are shown in Supplementary Tables S1–S2, with similar inverse associations.

**Table 2:** Weighted logistic regression analysis of LC9 and periodontitis prevalence

	Model 1 OR (95% CI)	p-value	Model 2 OR (95% CI)	p-value	Model 3 OR (95% CI)	p-value
LC9 (per 10 scores increase)	0.699 (0.663, 0.737)	<0.001	0.836 (0.789, 0.886)	<0.001	0.858 (0.808, 0.911)	<0.001
LC9 (quartile)						
Q1	Reference		Reference		Reference	
Q2	0.683 (0.588, 0.793)	<0.001	0.786 (0.650, 0.950)	0.012	0.818 (0.676, 0.990)	0.042
Q3	0.501 (0.428, 0.588)	<0.001	0.679 (0.564, 0.817)	<0.001	0.722 (0.601, 0.867)	<0.001
Q4	0.299 (0.238, 0.375)	<0.001	0.589 (0.466, 0.743)	<0.001	0.647 (0.509, 0.822)	<0.001
p for trend	<0.001		<0.001		<0.001	
Model 1: unadjusted model; Model 2: adjusted for age, sex, race, poverty-to-income ratio, education, marital status, drinking status; Model 3: adjusted for age, sex, race, poverty-to-income ratio, education , marital status, drinking status, flossing behavior, mouthwash behavior.

### Restricted Cubic Spline (RCS) Analysis

**Fig 2:**
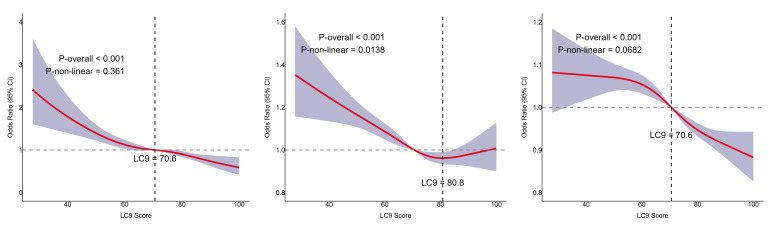

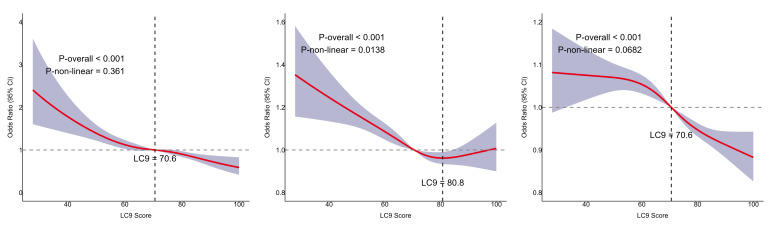
Nonlinear association between LC9 and periodontitis risk, CAL, and PD. a: Relationship between LC9 and periodontitis risk. b: Relationship between LC9 and PD. c: Relationship between LC9 and CAL.

RCS analysis indicated a linear inverse association between LC9 and both periodontitis risk and PD (p for nonlinearity = 0.361 and 0.0682, respectively; Figs 2a and 2c). In contrast, the LC9–CAL association exhibited a nonlinear trend, plateauing at higher LC9 scores (Fig 2b).

### Subgroup Analysis

**Fig 3:**
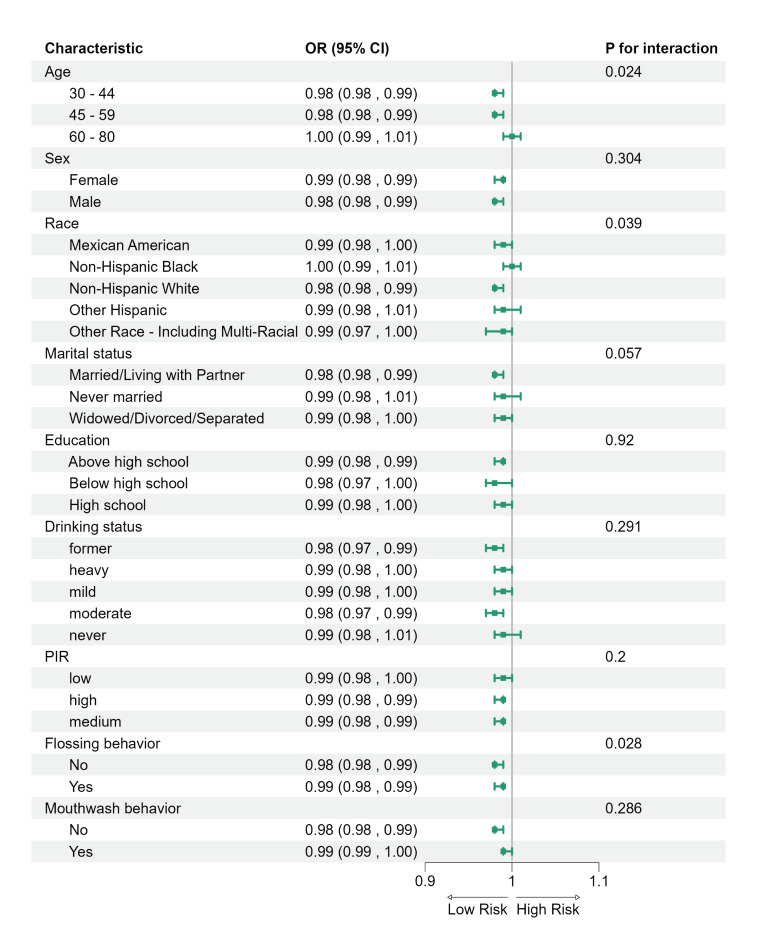
Stratified subgroup analysis of the association between LC9 and periodontitis in the NHANES population.

The inverse association between LC9 and periodontitis remained consistent across most subgroups (Fig 3). Statistically significant interactions were observed for age (p = 0.024), race/ethnicity (p = 0.039), and flossing behavior (p = 0.028). No statistically significant interactions were detected for sex, marital status, education, alcohol use, PIR, or mouthwash usage (all p > 0.05).

### Predictive Performance and Component Contribution

**Fig 4:**
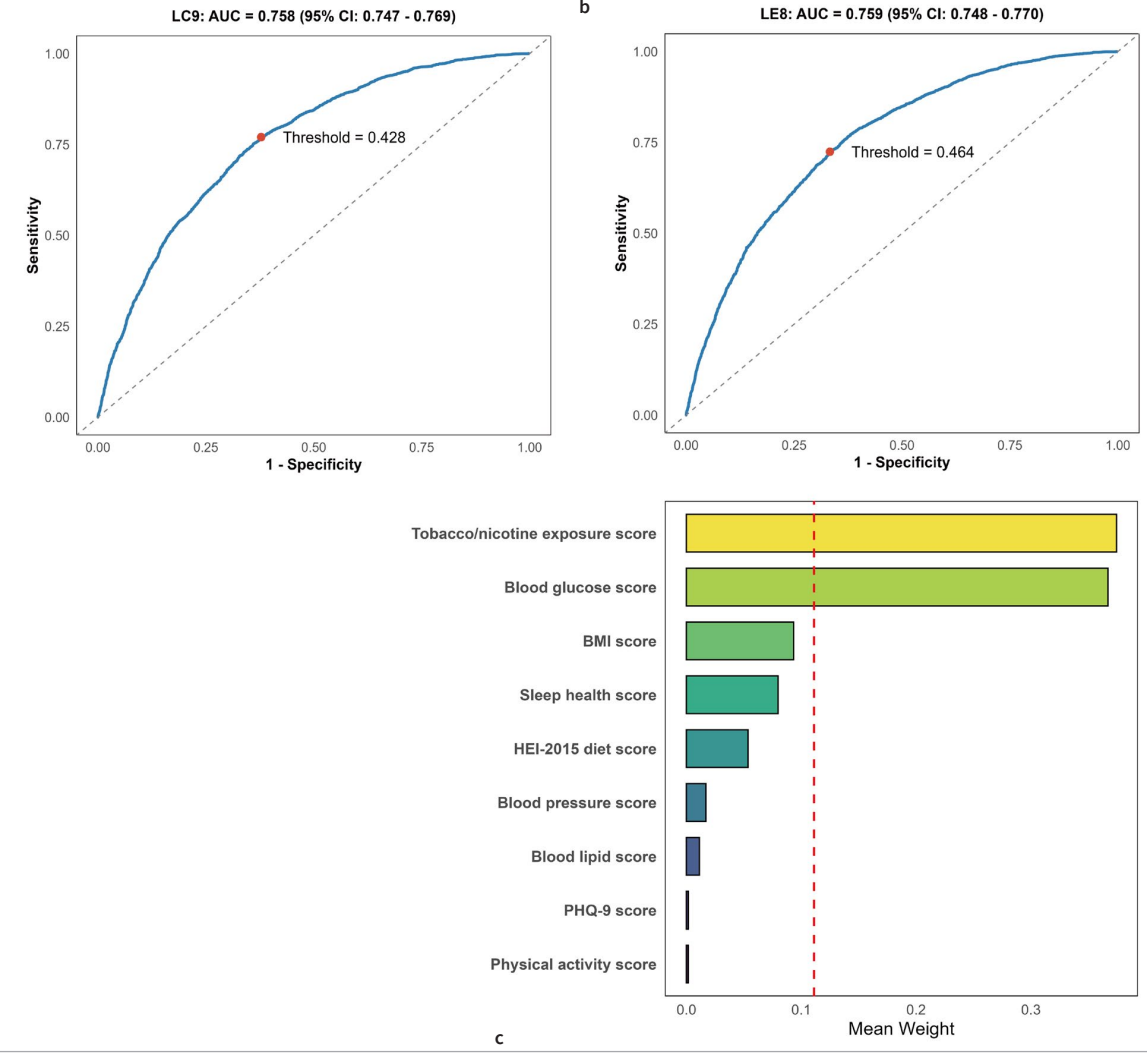
ROC curves and WQS regression weights for periodontitis prediction. a: ROC curve of LC9. b: ROC curve of LE8. c: Proportion of partial effect for each LC9 metric in the WQS regression. Model adjusted for age, sex, race, poverty-to-income ratio, education, marital status, drinking status, flossing behavior, and mouthwash behavior.

ROC analysis showed comparable predictive ability for periodontitis between LC9 (AUC 0.758, 95% CI: 0.747–0.769) and LE8 (AUC 0.759, 95% CI: 0.748–0.770) (Figs 4a–b). LC9 had slightly higher sensitivity (77.1% vs 72.4%), with similar specificity (62.2% vs 66.7%). WQS regression demonstrated a strong inverse association between the composite LC9 score and periodontitis risk (OR = 0.72, 95% CI: 0.68–0.77, p < 0.001). Tobacco exposure (weight = 0.3745) and glycemic control (weight = 0.3671) contributed most, while PHQ-9 had minimal influence, supporting the ROC findings (Fig 4c).

### Mediation Analysis

**Fig 5:**
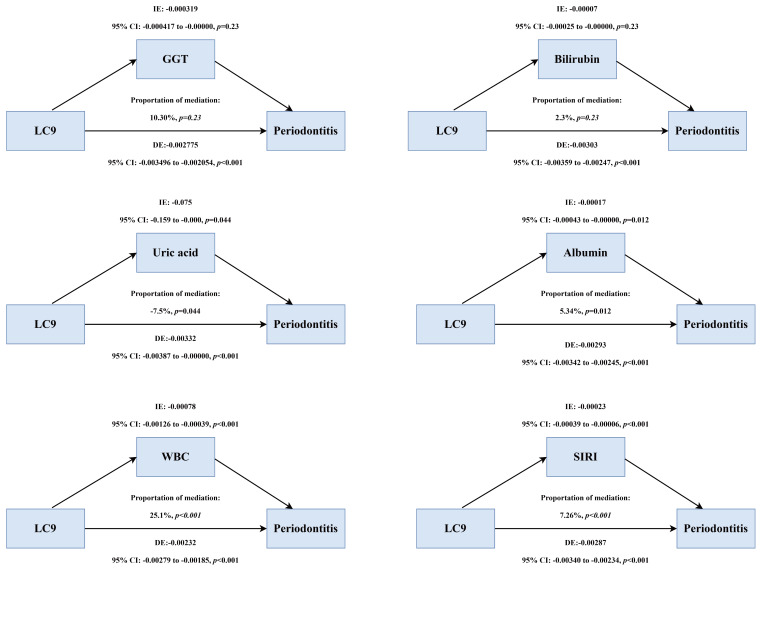
Schematic diagram of the mediation effect analysis. a: mediation of GGT. b: mediation of bilirubin. c: mediation of uric acid. d: mediation of albumin. e: mediation of WBC. f: mediation of SIRI.

Inflammatory markers statistically significantly mediated the LC9-periodontitis association: 25.1% for WBC, 7.26% for SIRI, and 5.3% for albumin (all p < 0.05). Among oxidative stress markers, GGT and bilirubin were not statistically significant, while uric acid showed a negative mediation effect (Fig 5).

## DISCUSSION

Drawing on nationally representative data from NHANES 2009–2014, this study investigated the association between the LC9 score—encompassing mental health—and the prevalence and severity of periodontitis. In addition to establishing a negative correlation between LC9 and periodontitis, we utilised RCS, WQS regression, and mediation analysis to thoroughly delineate the dose-response relationship, assess the relative contributions of each LC9 component, and explore potential biological pathways. Higher LC9 scores were statistically significantly associated with lower periodontitis risk, as well as reduced CAL and PD levels. The WQS analysis identified tobacco exposure and glycemic control as the most important factors, while mediation analysis indicated that systemic inflammation, specifically WBC and SIRI, served as the main pathway connecting LC9 to periodontal health. Conversely, oxidative stress markers had a negligible impact on this relationship.

These findings complement earlier NHANES-based studies on LE8 and periodontitis,^3,37^ and are consistent with recent evidence highlighting the broader relationship between periodontitis and systemic diseases.^14^ Additionally, depressive symptoms have been shown to correlate with periodontitis and may contribute to its progression through mechanisms such as inflammatory activation or reduced oral hygiene behavior.^12^ This study incorporated a psychological dimension into the LE8 framework for the first time, creating a more comprehensive LC9 score. We also systematically evaluated its association with multiple aspects of periodontitis, highlighting its potential utility as a tool for oral health assessment.

Interestingly, although LC9 integrates both behavioral and psychological dimensions, its predictive performance was only marginally better than LE8. Further WQS analysis revealed that the mental health component carried the lowest weight among the nine LC9 indicators, suggesting that its independent contribution to predicting periodontitis risk was relatively limited.This may be due to two reasons. First, the effect of depressive symptoms on periodontitis likely operates mainly through indirect pathways, such as inflammatory activation or changes in lifestyle, resulting in a weak direct effect in the model.^7^ Second, the psychological dimension may share substantial overlap with other components, such as sleep and physical activity, leading to multicollinearity and dilution of its unique explanatory power in multifactorial models.^8^

Therefore, health interventions in the general population should continue to prioritise high-weighted factors—particularly tobacco control and metabolic management—which have been consistently identified as the most important modifiable risk factors for periodontitis.^9,13,25^ Nevertheless, although the psychological dimension has limited weight in the overall population, its role in specific high-risk subgroups warrants further stratified analysis and validation.

The negative association between LC9 and periodontitis may primarily operate through mechanisms such as improved metabolic status and reduced systemic inflammation.^10,20^ Periodontitis, as a chronic low-grade systemic inflammatory condition, shares common inflammatory pathways with several chronic diseases, including atherosclerosis and insulin resistance.^11^

For the first time, mediation analysis was applied to examine the mechanistic relationship between LC9 and periodontitis. Systemic inflammatory markers, including WBC and SIRI, statistically significantly mediated the association, contributing 25.1% and 7.3% of the total effect, respectively. This finding is in line with recent evidence highlighting the critical role of inflammatory indices in linking periodontitis to systemic diseases, including cancer.^35^ In contrast, oxidative stress markers such as GGT and bilirubin did not demonstrate statistically significant mediation effects, suggesting their relatively minor contribution to the LC9–periodontitis pathway.

Although oxidative stress is commonly observed in both periodontitis and cardiovascular disease, it may act more as a downstream consequence of inflammation or be less sensitive to lifestyle-related interventions represented in LC9 components.^29,39^ Moreover, behavioral components of LC9—such as dietary habits, physical activity, and blood glucose regulation—appear to exert a more direct effect on systemic inflammation than on oxidative stress.^38^ As a result, the “exposure–mediator–outcome” chain may be attenuated for oxidative stress pathways.

Finally, although this study benefits from a large, nationally representative sample and rigorous analytical methods, certain limitations warrant caution. The cross-sectional design prevents causal inference, and the restricted periodontal measurements in NHANES may underestimate disease severity. Moreover, lifestyle and psychological variables were largely self-reported, introducing potential misclassification bias. Prospective studies with more comprehensive behavioral and biomarker assessments are needed to validate these findings.

## CONCLUSION

The LC9 score, a novel health assessment tool integrating lifestyle and mental health, shows potential value in assessing periodontitis risk and severity, with particular sensitivity in reflecting systemic inflammatory pathways. Clinically, promoting comprehensive lifestyle modifications—particularly tobacco cessation and glycemic control—could be incorporated into periodontal prevention and management strategies to effectively reduce disease risk and progression. Future studies should adopt longitudinal designs and include more comprehensive psychological and biological indicators to further elucidate the clinical significance and potential intervention pathways of LC9.

## ACKNOWLEDGMENTS

We appreciate the availability of the NHANES database, which offered valuable data resources essential to this research. This work was funded by the Provincial Science and Technology Program Project of Sichuan Provincial Department of Science and Technology (2022SNZY001), Science and Technology Project (Appropriate Technology Base) of Sichuan Provincial Health and Health Commission (2022JDXM021), Clinical Scientific Research Fund Project of the Chinese Stomatological Association of Western Stomatology (CSA-W2023-03).

### REFERENCES

**Table S1:** Weighted ordered logistic regression analysis of LC9 and periodontitis severity

	Model 1 OR (95% CI)	p-value	Model 2 OR (95% CI)	p-value	Model 3 OR (95% CI)	p-value
LC9 (per 10 scores increase)	0.705 (0.674, 0.737)	<0.001	0.852 (0.814, 0.892)	<0.001	0.859 (0.82, 0.90)	< 0.001
LC9 (quartile)						
Q1	Reference		Reference		Reference	
Q2	0.68 (0.592, 0.781)	<0.001	0.809 (0.68, 0.962)	0.017	0.825 (0.695, 0.979)	0.028
Q3	0.50 (0.433, 0.581)	<0.001	0.721 (0.616, 0.843)	<0.001	0.738 (0.63, 0.865)	<0.001
Q4	0.29 (0.238, 0.360)	<0.001	0.616 (0.502, 0.755)	<0.001	0.634 (0.516, 0.779)	<0.001
p for trend	<0.001		<0.001		<0.001	
Model 1: unadjusted model; Model 2: adjusted for age, sex, race, poverty-to-income ratio, education , marital status, drinking status; Model 3: adjusted for age, sex, race, poverty-to-income ratio, education, marital status, drinking status, flossing behavior, mouthwash behavior.

**Fig 1:** Flow chart of participants selection.

**Table S2:** Weighted linear regression analysis of LC9 and periodontal parameters

CAL	Model 1 Β (95% CI)	p-value	Model 2 Β (95% CI)	p-value	Model 3 Β (95% CI)	p-value
LC9 (per 10 scores increase)	-0.18 (-0.204,-0.156)	<0.001	-0.057 (-0.077,-0.038)	<0.001	-0.051 (-0.07,-0.033)	<0.001
**LC9 (quartile)**						
Q1	Reference		Reference		Reference	
Q2	-0.244 (-0.322,-0.167)	<0.001	-0.138 (-0.213,-0.063)	0.001	-0.121 (-0.194,-0.048)	0.002
Q3	-0.378 (-0.466, -0.290)	<0.001	-0.160 (-0.235,-0.085)	<0.001	-0.145 (-0.219,-0.070)	<0.001
Q4	-0.628 (-0.716,-0.541)	<0.001	-0.187 (-0.260,-0.113)	<0.001	-0.165 (-0.235,-0.095)	<0.001
p for trend	<0.001		<0.001		<0.001	
**PD**						
LC9 (per 10 scores increase)	-0.096 (-0.108,-0.084)	<0.001	-0.046 (-0.059,-0.033)	<0.001	-0.041 (-0.054,-0.028)	<0.001
**LC9 (quartile)**						
Q1	Reference		Reference		Reference	
Q2	-0.105 (-0.146, -0.065)	<0.001	-0.067 (-0.113, -0.021)	0.006	-0.055 (-0.101,-0.009)	0.021
Q3	-0.186 (-0.233, -0.139)	<0.001	-0.105 (-0.155, -0.056)	<0.001	-0.094 (-0.142, -0.045)	<0.001
Q4	-0.348 (-0.392, -0.304)	<0.001	-0.167 (-0.218, -0.117)	<0.001	-0.150 (-0.199, -0.101)	<0.001
p for trend	<0.001		<0.001		<0.001	
Model 1: unadjusted model; Model 2: adjusted for age, sex, race, poverty-to-income ratio, education, marital status, drinking status; Model 3: adjusted for age, sex, race, poverty-to-income ratio, education , marital status, drinking status, flossing behavior, mouthwash behavior.
